# The PDB and protein homeostasis: From chaperones to degradation and disaggregase machines

**DOI:** 10.1016/j.jbc.2021.100744

**Published:** 2021-05-04

**Authors:** Helen R. Saibil

**Affiliations:** Institute of Structural and Molecular Biology, Birkbeck, University of London, London, UK

**Keywords:** chaperone, protein folding, protein misfolding, protein aggregation, proteostasis, GroEL, DnaK, ATPases associated with diverse cellular activities (AAA), heat shock protein 90 (Hsp90), AAA, ATPases associated with diverse cellular activities, EM, electron microscopy, Hsp, heat shock protein

## Abstract

This review contains a personal account of the role played by the PDB in the development of the field of molecular chaperones and protein homeostasis, from the viewpoint of someone who experienced the concurrent advances in the structural biology, electron microscopy, and chaperone fields. The emphasis is on some key structures, including those of Hsp70, GroEL, Hsp90, and small heat shock proteins, that were determined as the molecular chaperone concept and systems for protein quality control were emerging. These structures were pivotal in demonstrating how seemingly nonspecific chaperones could assist the specific folding pathways of a variety of substrates. Moreover, they have provided mechanistic insights into the ATPase machinery of complexes such as GroEL/GroES that promote unfolding and folding and the disaggregases that extract polypeptides from large aggregates and disassemble amyloid fibers. The PDB has provided a framework for the current success in curating, evaluating, and distributing structural biology data, through both the PDB and the EMDB.

The elucidation of the heat shock response in the 1960s, showing that a set of characteristic changes in gene expression is triggered by environmental changes such as heat stress (1), led to the discovery of the molecular chaperones and the concept of protein quality control by Lindquist, Ellis, Craig, Hartl, Horwich, and many others. The regulation of protein synthesis, folding, and degradation is now known to be a central part of cell and molecular biology, and studies of the underlying mechanisms have been a rich source of new biological understanding.

However, the notion that a process as specific as the folding of a protein could be assisted by a relatively unselective helper protein with a broad range of different substrate proteins presented an intriguing structural and mechanistic puzzle. Protein structures and the PDB, directly and indirectly, have played a central role in tackling this puzzle and in the development of the chaperone field.

Structures of the key, general molecular chaperones, solved by X-ray crystallography and NMR spectroscopy, began to appear during the 1990s. These structures, including the first publications of Hsp70, GroEL, Hsp90, and small heat shock protein structures, were major events in the chaperone field and generated lots of excitement as they started to reveal the roles and actions of chaperones. The first structure was that of the ATPase domain of an Hsp70 (Fig. 1*A*, left). The structure was published in 1990, and the PDB entry (3hsc) appeared in 1995 (2). The big surprise was that Hsc70 ATPase domain had the same fold as actin and hexokinase. The actin structure, another milestone, was also published in 1990 ((3), in complex with DNase1, 1atn). Flexibly connected domains enclose the nucleotide binding cleft, with many sites for allosteric regulation on the outside, providing for multiple interaction partners with both actin and Hsp70. A structure of the substrate-binding domain, revealing the remarkable path of an extended substrate peptide threaded through a hole in the flat, brick-shaped domain with a movable lid, was published in 1996 ((4), Fig. 1*A*, right). But it would take until 2012 for the two domains to be captured together, tightly interacting in a dramatically changed conformation, instead of two separate structures loosely tethered by a flexible linker ((5), Fig. 1*B*). Opening and shutting of the substrate-trapping lid is allosterically coupled to the ATPase cycle (6). It is clear how trapping of extended segments in Hsp70 could maintain nascent chains in an extended state, *e.g.*, for translocation across an organelle membrane, but less obvious how this folds proteins. The general idea began to emerge that chaperones can use unfolding to assist folding, for example, by releasing kinetically trapped intermediates or by preventing premature formation of folding intermediates as nascent chains emerge from the ribosome or are being transported across organelle membranes.

**Figure 1:**
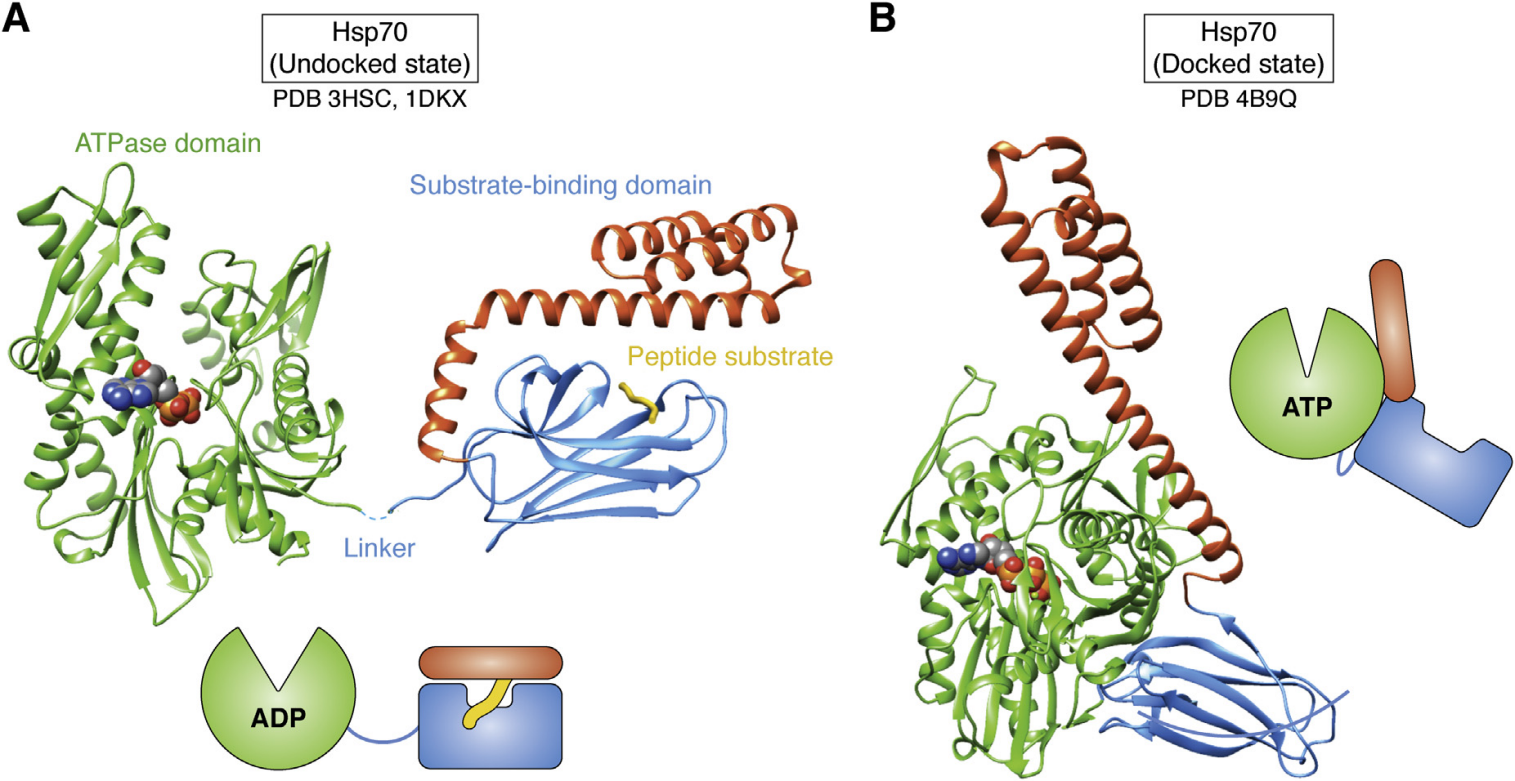
**Hsp70.***A*, the structures of the ATPase domain in *green* ((2), 3hsc) and the substrate-binding domain in *blue* and *orange* ((4), 1dkx). The presence of ADP in the nucleotide pocket favors the undocked state in which the two domains are separated by a flexible linker. A short peptide substrate (*yellow*) is shown threaded through its binding pocket. *B*, the ATP-bound, domain-docked state, in which the linker becomes ordered and the lid (*orange*) of the substrate domain is held open by the interaction with the ATPase domain ((5), 4b9q). Cartoons depict the undocked and docked states.

To explain the next set of developments, I will digress *via* some personal recollections. By the start of the 1990s, I had been recruited to the crystallography department at Birkbeck, where Steve Wood, working with Tom Blundell, was trying to crystallize a large oligomeric protein known as GroEL. Unlike most structural biologists at that time, Tom and Steve were enthusiastic about the idea of using electron microscopy (EM) to look at large complexes. We were still in the era when disdain was a typical reaction of crystallographers to “blobology,” if they had indeed even heard of using EM to look at macromolecular complexes. Soon after I set foot in the department, Steve told me about the large protein he was trying to crystallize that was somehow involved in protein assembly, and I was intrigued by the available EM images. I was easily persuaded to have a look, and from my first glimpse of it I was totally captivated. I felt sure that we could learn a lot from the images. The pioneering work on bacteriorhodopsin by Henderson and Unwin (7) had shown that it was possible to get 3D structures from EM images of macromolecular samples with some kind of ordered repeat. The interplay between crystallography and EM was becoming clear. Single particle EM was advancing for symmetrical viruses, and more slowly, but with far-reaching consequences, for asymmetric structures such as the ribosome. For GroEL, which has sevenfold symmetry, the structural changes were so dramatic when ATP and the cochaperone GroES were added, that they were obvious from the raw negative stain EM images. That excitement redirected my research, and having landed in a crystallography department, the PDB became part of my scientific environment.

In the case of GroEL, the structures told a lot of the story. For us, the first step was a low-resolution 3D EM map (8) that revealed a cage-like complex with internal cavities. It seemed very likely that something interesting would happen inside this cage. At that stage there was no database deposition for EM structures. We improved on this map in 1994 with relatively crude cryo EM maps showing substrate density in an open cavity and an enclosed space under the GroES (9). A month later came a big breakthrough with the crystal structure of GroEL by Braig *et al.* ((10), Fig. 2*A*). The structure showed that the internal cavities were lined by hydrophobic sites, and targeted mutagenesis showed the key role of the hydrophobic sites in substrate binding and folding (11). In 1996 we improved the EM maps, still at low resolution, but which nevertheless revealed a set of conformational changes triggered by nucleotide and GroES binding (12). Then came the structure of GroES and its mobile loops that provide flexible links to GroEL (13). This was followed by another big breakthrough from the Yale group—the crystal structure of a GroEL-GroES complex in 1997 ((14), Fig. 2*B*). The key elements of the mechanism were becoming clear: the nonnative protein was trapped on the hydrophobic lining of an open cavity, but then the combined actions of ATP and GroES binding caused a major reorganization of the complex so that the substrate was ejected from its binding sites but then trapped inside an enclosed cavity, capped by GroES, with a now hydrophilic lining—the folding chamber (Fig. 2*C*). This encapsulation, possibly following some forced unfolding during the dramatic restructuring of the complex (15), left the substrate protein with no choice but to remain the same or to collapse into its correct, native fold (Fig. 2*D*). Subsequent ATP hydrolysis allowed the release of the GroES lid and the contents of the folding chamber, whether folded or not. Nonnative protein would be recaptured for another round of interaction, whereas native protein would not incorrectly expose hydrophobic surface and would no longer bind to GroEL. At that point, the overall machine principle was clear—the nonnative protein would bind in an initially hydrophobic open cavity that would radically reorganize to trap the substrate in a hydrophilic space, perhaps after giving it some forceful tugs (15, 16).

**Figure 2:**
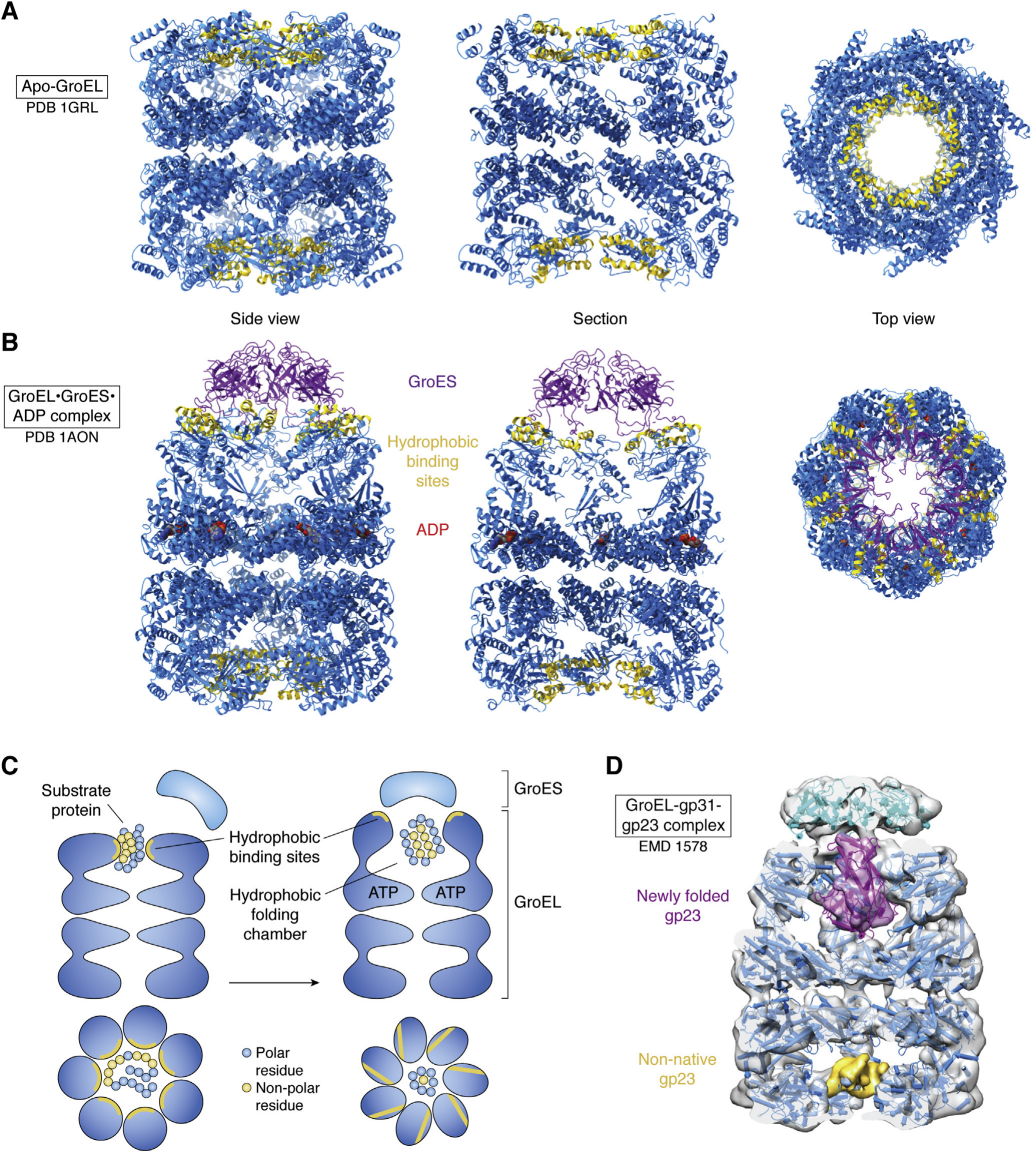
**GroEL-GroES and interactions with a nonnative substrate protein.***A*, the structure of apo GroEL, shown from the side, in a cut-away view and from the top ((10), 1grl). Helices H and I, which harbor many of the key hydrophobic substrate-binding sites (11), are shown in *yellow*. *B*, the equivalent representations of the GroEL-GroES-ADP complex, with GroES in *purple* and the nucleotide in *red* ((14), 1aon). *C*, cartoons showing GroEL side and top views with a partly unfolded protein captured on the hydrophobic binding sites (*yellow*) and then encapsulated and folded in the hydrophilic folding chamber of GroEL-GroES. *D*, section of a cryo EM map of GroEL bound to gp31, the bacteriophage T4 homologue of GroES, with a partly folded subunit of the T4 capsid protein gp23 (*pink*) inside the folding chamber, and partial density for a nonnative gp23 (*yellow*) in the open ring ((32), EMD-1548).

Still more chaperone structures were first revealed in the 1990s: Hsp90 occupies a central regulatory hub, interacting with many important biological pathways, particularly in signaling and the control of gene expression in development. Hsp90 is a dimer resembling a pair of cupped hands that open and close with its ATPase cycle and interactions with a wide range of cofactors. It is important in the activation of a somewhat more specific set of substrates, including signaling molecules such as steroid receptors and kinases, at a late stage of their folding. Its flexibility, particularly in the open forms, makes it a particularly difficult structural target. The first structures of domains began to appear in the late 1990s, but after 2000 different nucleotide bound states of the full dimer, often with bound cofactors, began to appear (Fig. 3). More recently, a full structure of a kinase substrate complex was solved, showing a remarkable split conformation with the two domains of the kinase stretched apart by a linker region running across the center of the Hsp90 complex (17).

**Figure 3:**
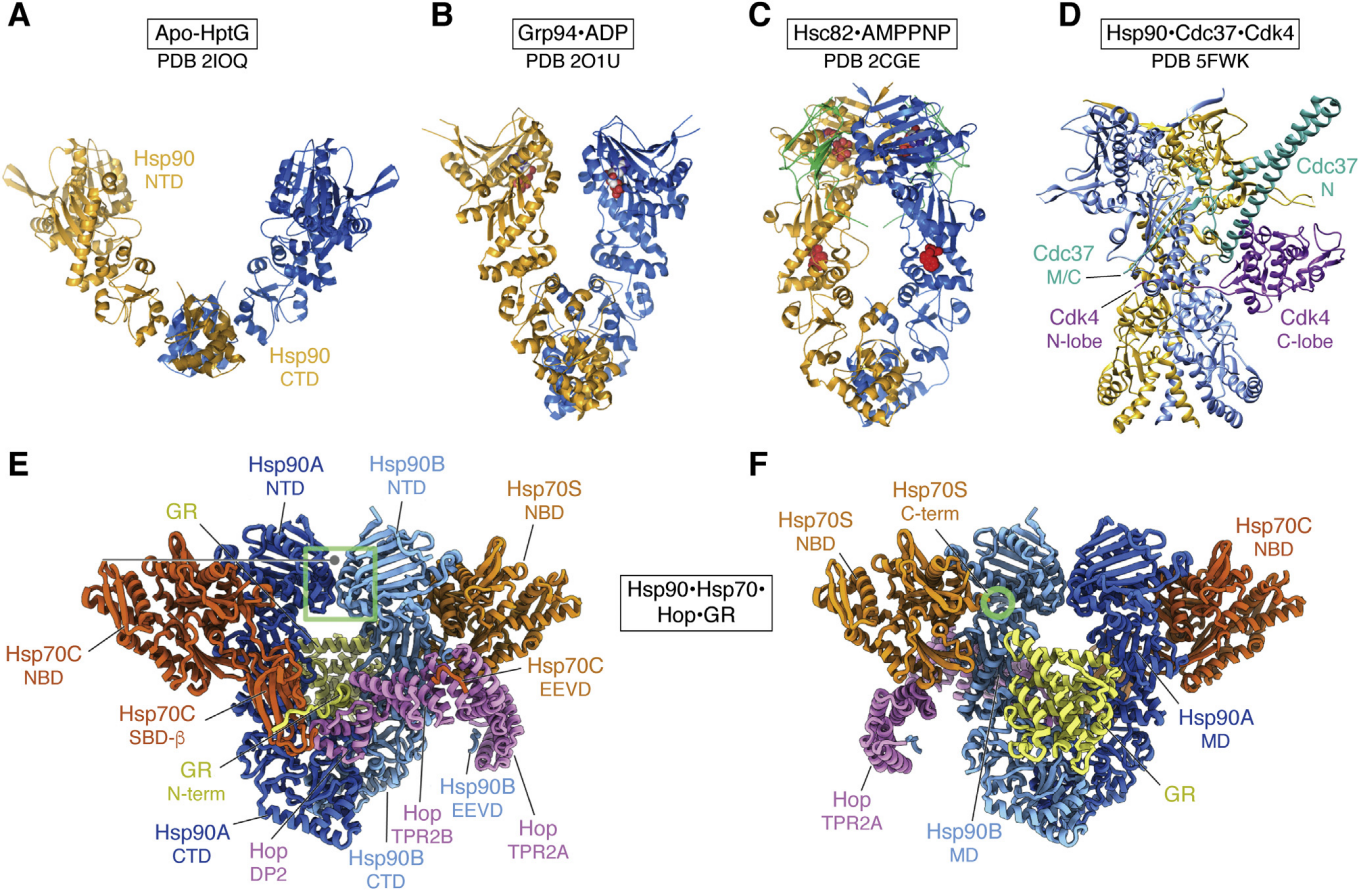
**Hsp90.***A*, the open, apo form of the bacterial Hsp90, HptG ((33), 2ioq), with one subunit in *blue* and the other in *gold*. *B*, the ADP-bound version of the ER form, Grp94 ((34), 2o1u). *C*, the closed, AMPPNP-bound state of the constitutive form Hsc82 ((35), 2cge). The *green* subunits are the two copies of cochaperone p23. *D*, the Hsp90 complex with co factor cdc37 (*green*) and kinase cdk4 substrate (*purple*) threaded through the complex with its N and C-terminal domains on opposite sides ((17), 5fwk). The linkers to the CDC37 C-terminal and CDK4 N-terminal domains are labeled. *E* and *F*, front and back views of the loading complex, formed of an Hsp90 dimer (*dark* and *light blue*), two Hsp70s (*orange*), the cochaperone HOP (*pink*), and the glucocorticoid receptor substrate (GR; *yellow*; (25)).

Also in the late 1990s, the first structure was determined of another class of abundant and widespread chaperones, the small heat shock proteins. The small Hsps are more mysterious mechanistically: they are not ATPases and form a wide variety of assemblies, ranging from monomers or dimers to 24-mers and higher oligomers. Some adopt regular, cage-like forms, such as the octahedral small Hsp from a thermophile, whose structure was determined by Kim in 1998 ((18), Fig. 4). They act in many biological settings, with many abundant forms in plants, and are significantly elevated in chronic inflammatory conditions. They provide a large capacity for reversibly binding nonnative proteins and thereby preventing aggregation, through a variety of interactions between ordered and disordered regions (19). They are thought to release their substrates for refolding by other chaperones, when stress conditions are relieved.

**Figure 4:**
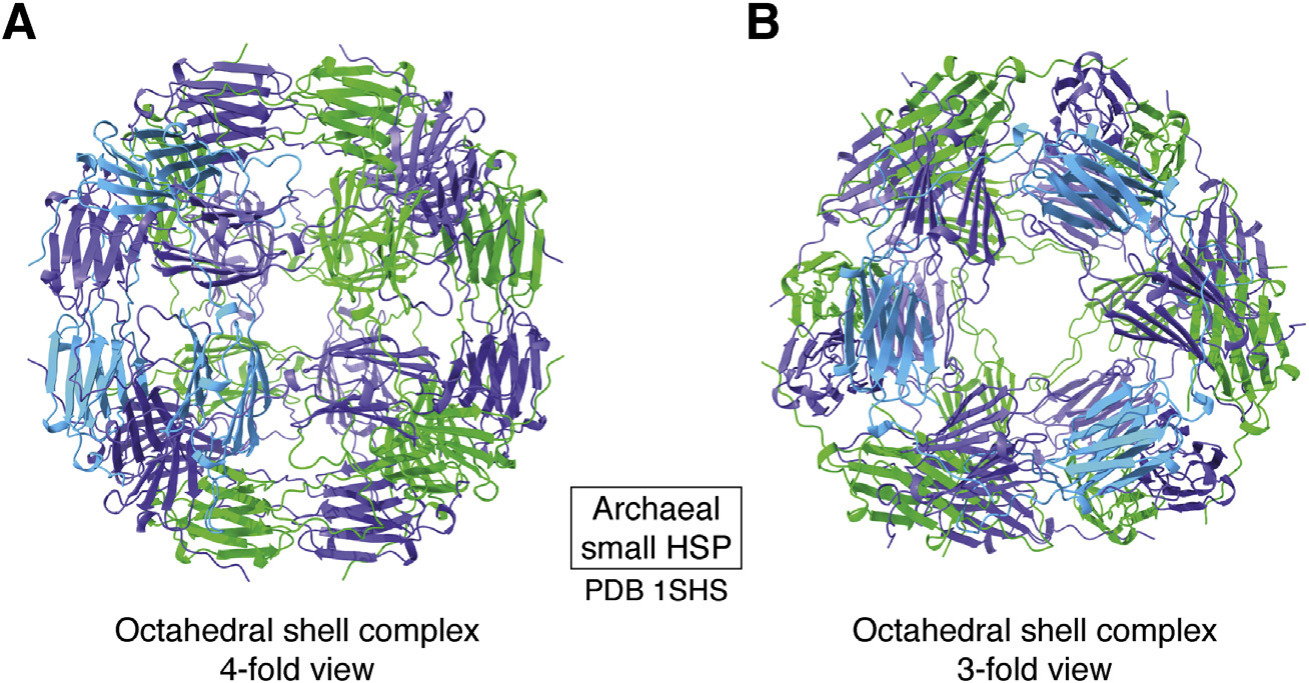
**Archaeal small HSP.***A*, view of the octahedral shell complex along its fourfold symmetry axis ((18), 1shs). *B*, view along the threefold axis.

In recent years, the focus for structural biologists interested in large macromolecular machines has shifted to cryo EM, as multiple advances combined to fuel an explosion of progress. As the resolution of single particle EM has improved, atomic models, built either *de novo* from high-resolution cryo EM maps or deduced from docking of previously known domains, have been arriving in the PDB in increasing numbers, now totaling over 9000 entries. During this development, the PDB and the field of protein crystallography in particular have provided a role model for the developing field of cryo EM. The organization created by the structural biology community with the PDB and the collaborative software project CCP4 (https://www.ccp4.ac.uk/) provided a path for organizing the scientific community, sharing data and software, and developing standards. The EM databank, EMDB, was based on the PDB and hosted at PDBe (https://www.ebi.ac.uk/pdbe/).

The 1990s saw the initial development of the EMDB with a European Union grant awarded to Carazo and Fuller (20). The current form of the EMDB was launched by Henrick at the European Bioinformatics Institute with further EU and EMBO support in 2002, and it is now closely coordinated with the PDB.

The early attempts by the EM community to use structures in the PDB were fraught with difficulties. In EM, the coordinate system puts the origin in a corner of the image or map. Crystallographic density maps place it at the center of the map. The first attempts to combine GroEL atomic coordinates and EM maps were amusing—every time someone tried to fit the coordinates into the map, the two things were so far apart that you couldn’t find them both on the display at the same time. They would literally disappear into a point if you zoomed out enough to get them both in the picture. Crystallographic and EM map formats had to be reunited as the two fields came back together after some decades of separate development, so that EM data could be read by the programs without causing software crashes. This reunification started during the 1990s. Now, the EM community has CCP-EM (https://www.ccpem.ac.uk/) to help with software support and training, and together with the databases, to establish data and validation standards.

The discovery of chaperones and the advances in understanding their actions in protein quality control has merged into the wider field of protein homeostasis or proteostasis. Chaperones are components of proteostasis networks that prevent or even reverse aggregation (21, 22). In addition, they regulate the flow of protein synthesis and degradation, with parallel protein quality control systems in the organelles (23). These actions support a wide range of physiological roles related to overall health and ageing, in addition to their assistance to protein folding and assembly. At the same time, the power of cryo-EM has rocketed forward to reveal at atomic resolution the mechanistic details of the cellular machinery performing these roles (*e.g.*, (24)). A recent, spectacular example of the cooperation between different chaperone systems is the cryo EM structure of the glucocorticoid receptor loading complex, in which the receptor is extended through Hsp90, also interacting with the cochaperone HOP and Hsp70, with a second Hsp70 scaffolding another part of HOP (Fig. 3, *E* and *F*; (25)).

It was initially thought that large aggregates, and particularly the amyloid fiber deposits seen in a broad class of protein misfolding diseases (26), were irreversible, dead-end states. But it has gradually emerged that certain chaperone systems, often multiple ones working together, can extract monomers from large aggregates, returning them to their soluble forms and effectively reversing aggregation (27). This changes our understanding of the progression of protein misfolding diseases, which likely involve an age-dependent, shifting balance between aggregation and disaggregation, leading to the eventual accumulation of aggregates in late stages of these diseases. In one class of disaggregases, found in bacteria, fungi, and plants, a subset of hexameric, AAA+ Hsp100 ATPases cooperate with the Hsp70 system to extract proteins from large aggregates and unfold them by threading through the central channel of the hexameric Hsp100 ring (Fig. 5, *A*–*C*; (28, 29)). Related Hsp100 proteins also function in ATP-dependent proteases such as the proteasome, where they unfold substrate polypeptides and thread them into the protease chamber (24). A different class of disaggregases, found in metazoa as well as other eukaryotes, is based on a specific version of the Hsp70 system, with the constitutive form of Hsp70, Hsc70, together with its cofactors DnaJB1 and the nucleotide exchange factor Apg2/Hsp110. This system has been shown to disassemble *in vitro* grown amyloid fibers of α-synuclein and mutant huntingtin (Fig. 5*D*; (30, 31)).

**Figure 5:**
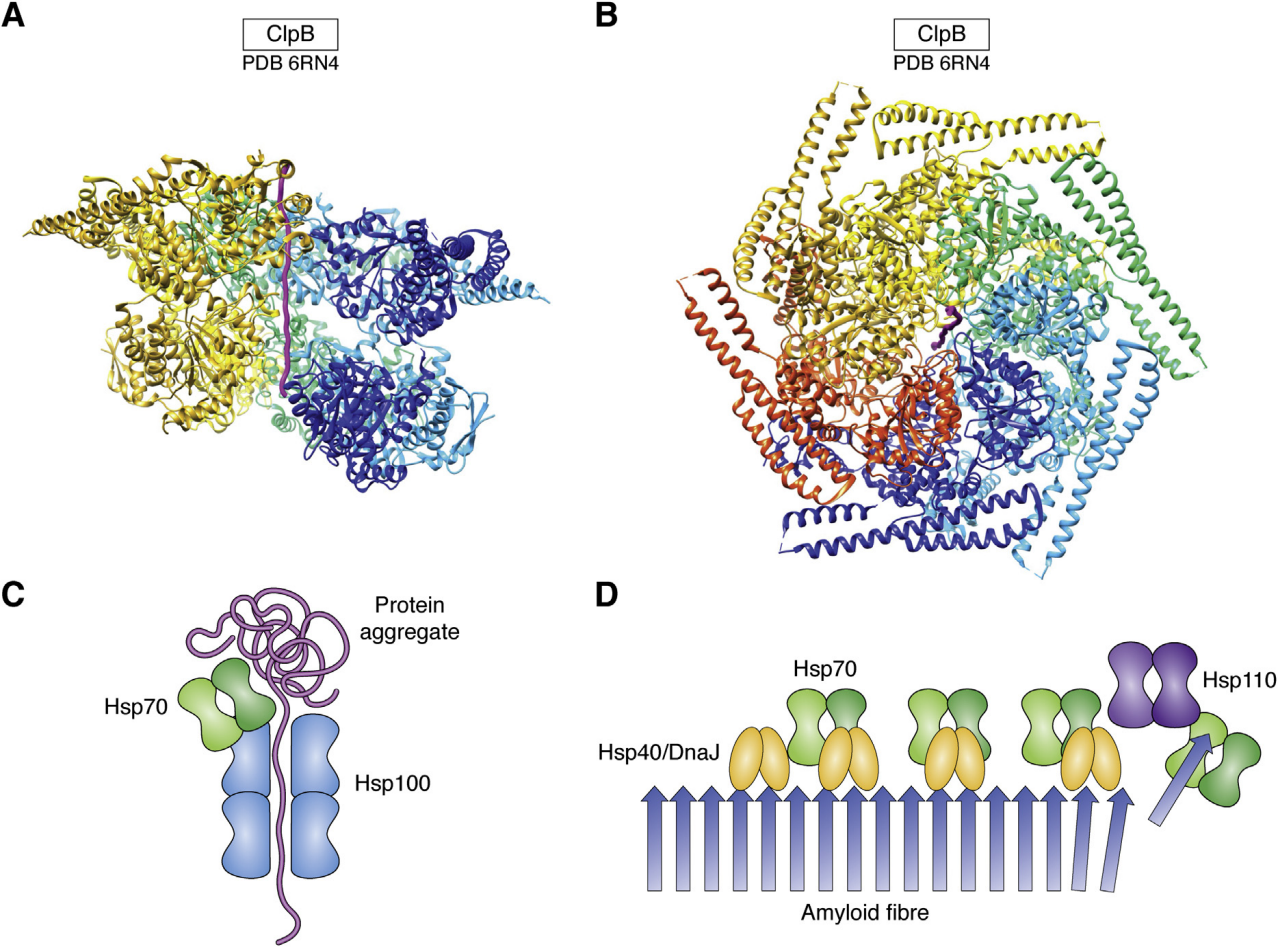
**An Hsp100 disaggregase threading a substrate polypeptide, and schematics of disaggregation.***A*, side view of the *E. coli* Hsp100, ClpB, threading the model substrate casein (*magenta*) through its central channel (29). The front subunit is removed to reveal the channel, and the complex is colored by subunit. *B*, top view of the ClpB hexamer, showing the coiled-coil regulatory domains that inhibit the ATPase and threading activity until they interact with Hsp70. This complex is in the repressed form, making inhibitory contacts at the tips of the coiled coils. *C*, cartoon of the protein remodeling ATPase Hsp100 (*cyan*) threading a polypeptide chain (*magenta*) being extracted from an aggregate with the cooperation of Hsp70 (*green*). *D*, cartoon of an amyloid fiber (*blue*) being disassembled from one end by the Hsp70-Hsp40/DnaJ-Hsp110/nucleotide exchange factor system (*green*-*orange*-*purple*).

Many areas of structural biology have been closely associated with the database/community developments described above, notably the field of protein synthesis, folding, and chaperones, which together provide the system for proteostasis, in addition to other major biological areas such as virology. The ability to share, reuse, and evaluate structural data is an integral part of the huge progress in structural biology.

## Conflict of interest

The authors declare that they have no conflicts of interest with the contents of this article.
